# A case of asymptomatic complete tracheal rings in an adult: case report

**DOI:** 10.1186/s40981-019-0265-7

**Published:** 2019-07-12

**Authors:** Tomoko Hayasaka, Takafumi Kobayashi, Yoshida Ako, Yasuhiro Endo, Yuko Saito

**Affiliations:** 1 Tohoku Rosai Hospital, 4-3-21 Dainohara Aoba-ku Sendai-shi, Miyagi, Japan; 2 Osaki Citizen Hospital, 3-8-1 Furukawa Homami Osaki-shi, Miyagi, Japan; 3 Tohoku University Hospital, 1-1 Seiryo-chou Aoba ku Sendai-shi, Miyagi, Japan

To the Editor,

Complete tracheal rings are a rare congenital defect that can cause tracheal stenosis. There are only a few reports of symptomatic adult cases, with many associated with difficult intubation [1, 2]. We report a case of a patient with complete tracheal cartilage rings without symptoms or histories of difficult intubation.

The patient was a 70-year-old man with right arteriosclerosis obliterans (ASO). He had also undergone a right iliac artery stenting procedure for ASO at age 67 under general anesthesia without difficult intubation. Left external iliac artery stenting and femoral endarterectomy were scheduled. After anesthesia was induced, direct laryngoscopy was conducted using a Macintosh laryngoscope. We attempted tracheal intubation but were unable even with an endotracheal tube of 7.0 mm (Parker®, Parker Medical, Highlands Ranch, CO, USA), due to subglottic resistance. A laryngeal mask airway (i-gel® #4, Intersurgical Ltd., Liverpool, NY, USA) was then utilized to provide airway management, and the surgery was performed as scheduled. Intraoperative bronchoscopy showed complete tracheal rings with the absence of the membranous trachea immediately below the cricoid cartilage to the tracheal bifurcation. Normal membranous tissue was confirmed below the bifurcation (Fig. 1).

**Fig. 1.**
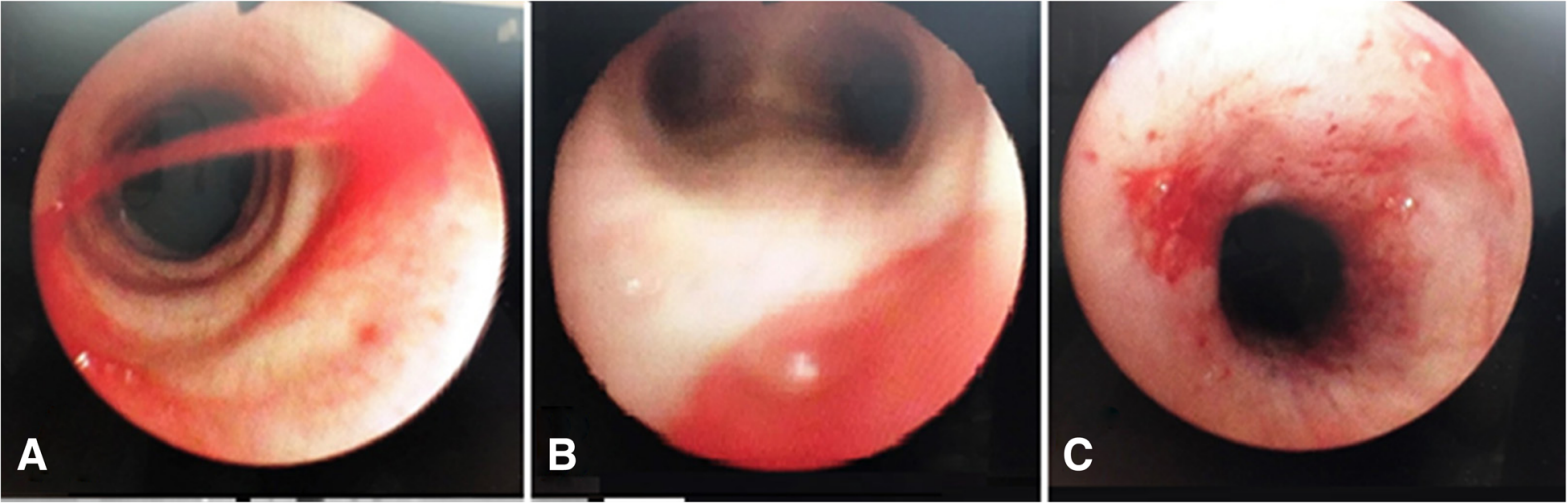
Bronchoscopy photos. **a** Complete tracheal rings with the absence of the membranous portion of the trachea immediately below the cricoid cartilage to the tracheal bifurcation were observed. **b** Membranous tissue was noted at the bifurcation. **c** Petechiae of the mucosa immediately below the cricoid cartilage was observed

On CT imaging (Fig. 2), the trachea was observed as specific O shape and the inner tracheal diameter at the site of the complete rings was greater than the outer diameter of the 7.0-mm endotracheal tube. The coronal section of multiplanar reconstruction CT showed the trachea as an upside-down bottle neck shape and revealed complete tracheal rings narrowing the trachea 21 mm caudad to the vocal cord. Although the transverse diameter at the site of transition to the tracheal rings was relatively smaller than at the cricoid cartilage level, there was no significant stenosis to cause respiratory symptoms.

**Fig. 2.**
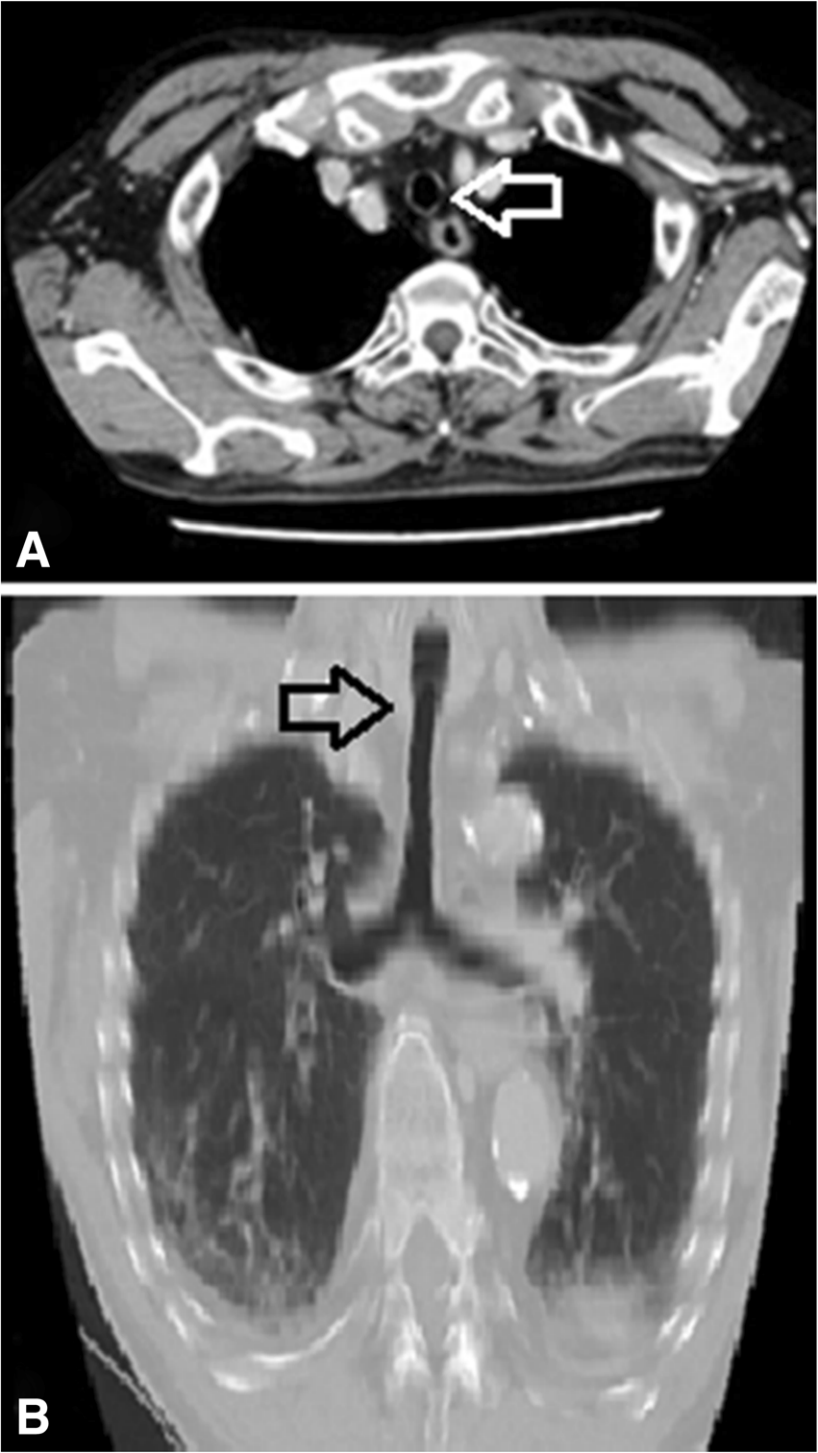
CT imaging of complete tracheal rings. **a** Preoperative axial CT imaging of tracheal rings (arrow). **b** Postoperative coronal CT reconstruction (arrow). The tracheal diameter at the site of transition to complete tracheal rings was slightly smaller than at the cricoid cartilage level. However, there was no significant stenosis

Complete tracheal rings are usually detected in the neonatal period or infancy as congenital tracheal stenosis, with symptoms of stridor, cyanosis, retractive breathing, or suffocation. Cases diagnosed in adulthood are extremely rare. There have been only 13 reported cases of complete tracheal rings with tracheal stenosis discovered in adults; of these, seven were found only when intubation failed [2]. Boiselle et al. suggested that thoracic CT images can be used to diagnose tracheal rings as concentric narrowing of the trachea with an O-shaped lumen [3]; contrarily, the normal trachea appears C-shaped.

In our case, although a tracheal tube could not be passed below the vocal cords, intubation had been successful at the previous general anesthesia. The tip of the tracheal tube was probably impinged at a caudad end of the normal trachea which was transition zone to the tracheal rings. To date, there have been no reports of cases of complete tracheal rings without symptoms such as tracheal stenosis and history of difficult intubation. This case suggests that the complete tracheal rings may be hidden even in normal adults for whom there has been no trouble with intubation. Multiplanar reconstruction CT helps to assess difficult intubation.

## Data Availability

Not applicable.

## Abbreviation

ASO: Arteriosclerosis obliterans
